# A Preliminary Comparison on Faecal Microbiomes of Free-Ranging Large Baleen (*Balaenoptera musculus*, *B. physalus*, *B. borealis*) and Toothed (*Physeter macrocephalus*) Whales

**DOI:** 10.1007/s00248-021-01729-4

**Published:** 2021-03-21

**Authors:** Stefanie P. Glaeser, Liliana M. R. Silva, Rui Prieto, Mónica A. Silva, Angel Franco, Peter Kämpfer, Carlos Hermosilla, Anja Taubert, Tobias Eisenberg

**Affiliations:** 1 Institute of Applied Microbiology, Justus Liebig University Giessen, IFZ-Heinrich-Buff-Ring 26-32, 35392 Giessen, Germany; 2 Institute of Parasitology, Justus Liebig University, Giessen, Germany; 3 Institute of Marine Research (IMAR) and Okeanos R&D Centre, University of the Azores, Horta, Portugal; 4 MARE—Marine and Environmental Sciences Centre, Lisbon, Portugal; 5 Department of Veterinary Medicine, Hessian State Laboratory (LHL), Giessen, Germany

**Keywords:** Large whales, Faecal microbiome, *Balaenoptera*, *Physeter*

## Abstract

Large baleen and toothed whales play crucial ecological roles in oceans; nonetheless, very little is known about their intestinal microbiomes. Based on striking differences in natural history and thus in feeding behaviours, it can be expected that intestinal microbiomes of large baleen whales and toothed whales are different. To test this hypothesis, the phylogenetic composition of faecal microbiomes was investigated by a 16S rRNA gene amplicon sequence–based approach for *Bacteria* and *Archaea*. Faecal samples from free-ranging large whales collected off the Azores Archipelago (Portugal) were used, comprising 13 individual baleen whales (one sei, two blue and ten fin whales) and four sperm whales*.* The phylogenetic composition of the *Bacteria* faecal microbiomes of baleen and toothed whales showed no significant differences at the phylum level. However, significant differences were detected at the family and genus levels. Most abundant phyla were *Firmicutes*, *Bacteroidetes*, *Proteobacteria*, *Tenericutes* and *Spirochaeta.* Few highly abundant bacterial genera were identified as key taxa with a high contribution to differences among baleen and toothed whales microbiomes. Only few archaeal sequences were detected, primarily *Methanomassiliicoccales* representing potential methanogenic *Archaea*. This is the first study that directly compares the faecal bacterial and archaeal microbiomes of free-ranging baleen and toothed whales which represent the two parvorders of Cetacea which members are fully aquatic large mammals which were evolutionary split millions of years ago.

**Supplementary Information:**

The online version contains supplementary material available at 10.1007/s00248-021-01729-4.

## Introduction

Baleen (parvorder *Mysticeti*) and toothed (parvorder *Odontoceti*) whales play essential roles in ocean ecosystem as apex predators, primary and secondary consumers and are, therefore, suitable as indicators of ocean health worldwide [1, 2]. Despite their ecological relevance, anthropogenic pressure has driven various large whales to endangered status, and even to near extinction by hunting, and overall degradation of marine environments [1–4].

Baleen and toothed whales comprise the two cetacean parvorders within the order Cetartiodactyla, suborder Cetacea, summing 89 extant species (14 and 75, respectively). Time-calibrated molecular phylogeny suggests that they split around 36 million years ago, and this divergence was in part driven by evolutionary innovations in feeding mechanisms [5–7]. Toothed whales evolved innovations combining echolocation and anatomical modifications, to target single prey items by raptorial biting or suction feeding, while baleen whales evolved keratinous plates (baleen) and modifications to the skull and mouth morphology, allowing bulk feeding on aggregations of small prey using filtration [6]. These specialisations resulted in a marked trophic separation between baleen (mean trophic level: 3.35) and toothed (mean trophic level: 4.23) whales, despite all being carnivorous [8, 9]. Taking these differences into account, one would expect striking differences in the two phyla also with respect to faecal microbiomes. This co-evolution of commensal/mutualistic microbes within marine vertebrate hosts may have had a strong contribution to their nutritional adaptation to specific diets [10]. Among a broad range of environmental factors and host genetics/phylogeny, diet composition (herbivore, omnivore or carnivore) is an essential factor in the acquisition and maintenance of a healthy intestinal microbiome, both in terrestrial and marine mammals [1, 11, 12]. Hence, largely different intestinal microbiomes can be expected in baleen and toothed whales.

The faecal microbiome as a proxy for vertebrate intestinal microbiome contains a high diversity of different microbes of multiple taxa and with different metabolic capacities including transient opportunistic microbes, food-borne communalists and often host-specific mutualistic microbes [10]. Especially the latter often play a critical role in the processing of food uptake but can also affect host metabolism, host development and immune system functioning [10]. In healthy individuals, intestinal microbiota are robust and resistant to perturbations and maintain their composition in physiological ranges in order to sustain homeostasis, symbiosis and development of proper innate and adaptive immunity [13].

Nonetheless, there are only a few studies focussing on faecal microbiomes of whales. Sanders et al. [14] performed a comprehensive study on the diversity and function of the faecal microbiome of baleen whales. In a phylogenetic diversity study based on 454 amplicon-generated 16S rRNA gene sequence libraries and generated shotgun metagenomes of selected samples, functions of the intestinal microbiome (total gene pool) of a sei whale (*Balaenoptera borealis*), seven right whales (*Eubalaena glacialis*) and four humpback whales (*Megaptera novaeangliae*) were studied. These samples were drawn from captured live whales or from faecal samples from the intestines of stranded carcasses. The faecal microbiomes of these baleen whale species were compared with those of captured belugas (*Delphinapterus leucas*) and several carnivorous or herbivores terrestrial mammals. The baleen whale microbiomes shared different functional traits either with faecal microbiomes of terrestrial carnivores, and on the other hand with those of terrestrial herbivores. Despite functional parallels of whale faecal microbiomes to those of terrestrial carnivores and herbivores, the phylogenetic composition of the faecal microbiome of the baleen whales sampled by Sanders et al. [14] was unique and clearly distinct to terrestrial species but also distinct to captured beluga whales (toothed whales).

There are only few other intestinal/faecal microbiome studies of other large marine mammals. Bik et al. [1] studied the faecal microbiomes (swab samples) compared to four other body sites of healthy dolphins and sea lions. They found that habitat, diet and the host phylogeny contributed to a specific composition of the intestinal microbiome. In another study of the faecal microbiome of bottlenose dolphins, Soverini et al. [15] compared their data to those of Sanders et al. [14] and concluded that the captive bottlenose dolphin intestinal microbiome was distinct from that of baleen whales, which they attributed to differences in the dietary niches. Furthermore, the authors indicated that the intestinal microbiome of dolphins showed a compositional similarity to intestinal microbiomes of carnivorous fishes and other marine piscivores, in contrast to earlier results on baleen whales [14].

Few other studies investigated the gut microbiome of baleen or toothed whales. Those studies included the investigation of the intestinal microbiome of stranded (dead) adult pygmy (*Kogia breviceps*) and dwarf (*K. sima*) sperm whales [16], southern right whale (*Eubalaena australis*) calves (baleen whales) [17], two free-ranging blue whales (*B. musculus*; baleen whales) [18] and different gut sections of 38 hunted bowhead whales (*Balaena mysticetus*, baleen whales) [19]. A direct comparison of toothed and baleen whale microbiomes studied in parallel was never performed.

Here we compared the faecal microbiomes of living, free-ranging, presumably healthy baleen whales of the family *Balaenopteridae* and of toothed whales (sperm whales *Physeter macrocephalus*), thereby analysing members of both whale phyla for the first time. Furthermore, sampling design was in full agreement with physiological defecation and did not induce any stress to these marine mammals or microbiome artefacts compared to stranded whales. For a comprehensive overview and unlike to most previous studies, our investigation also included assessment of *Archaea* as a separate prokaryotic group in 17 members of the *Cetartiodactyla* for the first time. The abundance and phylogenetic diversity of bacterial and archaeal members of faecal microbiomes were investigated by 16S rRNA gene target–based real-time PCR (qPCR) and 16S rRNA gene sequence–based Illumina amplicon sequencing using established *Bacteria-* and *Archaea*-specific primer systems.

## Material and Methods

### Sample Collection

Faecal samples of 13 baleen whales of the family *Balaenopteridae*, including one sei whale (*Baleaenoptera borealis*), two blue whales (*B. musculus*) and ten fin whales (*B. physalus*), and of four sperm whales (*P. macrocephalus*) were collected in 2011 and 2014 off the Azores Archipelago (Portugal; Supplementary Fig. S1; Supplementary Table S1). Samples were collected under research licences #51/2011/DRA and #20/2013/DRA granted by the Regional Government of the Azores. Collection methods have been described in Hermosilla et al. [20]. Briefly, samples were obtained during focal follows of individual whales considered healthy from visual inspection in the field. Animals were not disturbed. Upon defecation, samples were collected as quickly as possible using a mesh-dip net with a 400-μm mesh size. Approximately 10–50 ml of each faecal sample were transferred to sterile plastic Falkon vials and fixed with ethanol at 70% concentration and stored on ice. Upon arrival in the laboratory, samples were stored at 4 °C until being transferred to the Institute of Parasitology (Justus Liebig University, Giessen, Germany). For long-term storage, samples were stored at − 20 °C.

### DNA Extraction from Whale Faecal Samples

For DNA extraction, cooled faecal samples in 70% ethanol were pelleted by centrifugation (13,780*g* for 20 min at 4 °C) and washed twice with 1x autoclaved phosphate-buffered saline (PBS; 130 mM NaCl, 7 mM Na_2_HPO_4_, 3 mM NaH_3_PO_4_ per litre; pH 7) to remove remaining ethanol. Approximately 150 mg faecal samples were extracted with the ZR Faecal DNA MiniPrep Kit (Zymo Research Europe GmbH, Freiburg, Germany) as described by the manufacturer. DNA was finally eluted with 100 μL Ultra Pure™ demineralised water (DNase, RNase Free; Invitrogen, Karlsruhe, Germany) from the final spin column and measured in a Nano drop (Peqlab, Erlangen, Germany). The DNA concentration was adjusted to a concentration of 10 ng μL^−1^ for molecular biological analysis. The quality of the DNA samples was checked by PCR amplification of 16S rRNA gene fragments using universal *Bacteria* 16S rRNA gene–targeting primers and subsequent denaturing gradient gel electrophoresis (DGGE) analysis according to Schellenberg et al. [21]. All samples gave good amplification products (Supplementary Fig. S2a) and specific bacterial community fingerprint patterns (Supplementary Fig. S2b) which had already indicated significant differences among bacterial communities of baleen and toothed whale samples (Supplementary Fig. S3).

### Quantification of Total *Bacteria* and *Archaea* 16S rRNA Gene Targets in Faecal Samples

Quantitative PCR (qPCR) analysis was performed with the SsoFast™ EvaGreen® Supermix (Bio-Rad, Feldkirchen, Germany) in a total volume of 10 μL including 1 μL template DNA, 1× SsoFast™ EvaGreen® Supermix, 0.2 μM of *Bacteria* (Univ-F, 5´-GTGSTGCAYGGYTGTCGTCA-3´, Univ-R, 5´-CCCCTCKGSAAAGCCTTCTTC-3´; [22]) and 0.5 μM of *Archaea* (ARC787F, 5´-ATTAGATACCCSBGTAGTCC-3´, ARC1059R, 5´-GCCATGCACCWCCTCT-3´; [23]) 16S rRNA gene targeting primer sets. A serial dilution of 16S rRNA gene fragments in defined concentrations (1 × 10^2^ to 1 × 10^8^ targets μL^−1^) was used as DNA standard for the qualification of bacterial and archaeal 16S rRNA gene targets. The standards were generated by the amplification of the 16S rRNA gene from reference strains, *Citrobacter freundii* ATCC 8090^T^ (*Bacteria*) and *Saccharolobus solfataricus* P2 (*Archaea*) using universal primers as described by Cifuentes et al. [24]. The PCR products were purified (PCR purification kit; Qiagen) and quantified in a Tecan GENios FL fluorescence reader (Tecan Group Ltd., Männedorf, Switzerland) using PicoGreen (Quant-iT PicoGreen dsDNA reagent; Invitrogen, Germany) and a dilution series of Lambda DNA (Thermo Scientific, Germany) for quantification. The concentration of 16S rRNA gene targets μL^−1^ in the applied standards was calculated as described by Kolb et al. [25]. QPCRs were performed in a CFX96 Touch™ Real-Time PCR Detection System (Bio-Rad) by the use of the following PCR program: 98 °C 2 min, followed by 35 cycles of 98 °C 20 s, 60 °C 20 s and 72 °C 20 s (detection), followed by melting curve analysis by heating from 65 to 95 °C (+0.5 °C/0.5 s cycle). Samples were run in technical triplicates and standards in technical duplicates. Primer dimers were not detected. Gene copy numbers and qPCR efficiencies were calculated using the Bio-Rad CFX Manager software (version 3.0). Efficiencies of qPCRs were 94.6% (*R*^2^ = 0.996) for the *Bacteria* and 77.6% (*R*^2^ = 0.992) for the *Archaea* primer system, respectively. Significant differences among samples were tested in SigmaPlot v12.5 (Systat Software, Erkrath, Germany) by one-way analysis of variance (ANOVA) using the Tukey test (post hoc test), the Shapiro Wilk normality test and the Brown Forsythe equal variance test. Concentrations of bacterial and archaeal 16S rRNA gene targets in baleen whales (all species summarised) compared to toothed whales were tested by *T*-tests assuming unequal variances. Tests were performed in Microsoft Excel (version 16.16.27).

### *Bacteria* and *Archaea* 16S rRNA Gene Amplicon Sequencing Using an Illumina MiSeq Platform

The phylogenetic composition of bacterial and archaeal communities was analysed by 16S rRNA gene amplicon sequencing using universal 16S rRNA gene targeting primer systems, 341F (5´-CCTACGGGNGGCWGCAG-3´) and 785F (5´-GACTACHVGGGTATCTAAKCC-3´) for *Bacteria* and A340F (5´-CCCTAYGGGGYGCASCAG-3´) and A915R (5´-GTGCTCCCCCGCCAATTCCT-3´) for *Archaea*, respectively. Applied primer pairs were recommended by Klindworth et al. [26] for microbiome studies. PCR amplifications and Illumina 300 bp paired-end read sequencing using an Illumina MiSeq V3 system was performed by LGC Genomics (Berlin, Germany). The Illumina bcl2fastq 1.8.4 software (folder “RAW”) was used for dedublexing of all libraries. Reads were sorted by amplicon inline barcodes allowing one barcode mismatch. Reads with missing barcodes, one-sided barcodes or conflicting barcode pairs were discarded. Sequence adaptors were clipped in the following step and all reads with a length < 100 bp were discarded (adaptor clipping). Subsequently, primers (3 mismatches were allowed) were detected used for sequence orientation and clipped. Forward and reverse reads were combined using BBMerge 34.48 (http://bbmap.sourceforge.net/). The combined read pair data set was used for further analysis. Fastqc files of the combined reads were converted to fasta files using Galaxy (https://usegalaxy.org/) and submitted to the NGS analysis pipeline of the SILVA rRNA gene database project (SILVAngs 1.3; [27]). Each read was aligned using the SILVA Incremental Aligner (SINA; SINAv1.2.10 for ARB SVN [revision 21008]; [28]) against the SILVA SSU rRNA SEED and quality control [27]. Reads shorter than 50 aligned nucleotides and reads with more than 2% of ambiguities or 2% of homopolymers, respectively, were excluded from further processing. Putative contaminations, artefacts and reads with a low alignment quality (50 alignment identity, 40 alignment score reported by SINA) were excluded from downstream analyses. After these initial quality controls, identical reads were identified (dereplication), the unique reads (operational taxonomic units, OTUs) clustered on a per sample basis and the reference read of each OTU was classified based on the SILVA Taxonomy down to the genus level. Dereplication and clustering was done using cd-hit-est (version 3.1.2; http://www.bioinformatics.org/cd-hit;[29]) running in accurate mode, ignoring overhangs and applying identity criteria of 1.00 and 0.98, respectively. The classification was performed by a local nucleotide BLAST search against the non-redundant version of the SILVA SSU Ref dataset (release 128; http://www.arb-silva.de) using blastn (version 2.2.30+; http://blast.ncbi.nlm.nih.gov/Blast.cgi) with standard settings [30]. The classification of each OTU reference read was mapped onto all reads that were assigned to the respective OTUs and identical reads. Reads of all OTUs assigned to the same taxonomic paths were summarised for subsequent analysis. This yields quantitative information (number of individual reads per taxonomic path) within the limitations of PCR and sequencing technique biases as well as multiple rRNA operons. Reads without any BLAST hits or reads with weak BLAST hits, where the function “(% sequence identity + % alignment coverage)/2” did not exceed the value of 93, remained unclassified and were assigned to the meta group “No Relative” [31]. The applied method was first used in the publications of Klindworth et al. [26] and Ionescu et al. [32]. For relative abundance analysis of bacterial communities reads assigned as *Archaea*, mitochondria, chloroplasts, Eukarya or “No Relative” were excluded for the analysis of bacterial faecal microbiome. *Bacteria-*derived sequences were set to 100% for further analyses. Bacterial communities were analysed at three taxonomic path levels, phylum, family and genus. Due to the limited resolution of the 16S rRNA gene (genus level) subsequent analyses were not performed at the OTU level. Total sequence numbers per taxonomic paths and relative abundance patterns of the resulting bacterial community profiles (resolution: phyla, families, phylogenetic groups) were further used for detailed analysis in PAST4 (folk.uio.no/ohammer/past; [33]). Absolute sequence numbers per taxonomic path (genus level) were used for alpha diversity analysis including the Chao-1 index (estimating the number of present genera/phylogenetic groups), evenness and dominance values (describing the number and distribution of the phylogenetic groups within the microbiomes), and the Shannon diversity index (describing the overall taxa diversity), as well as rarefaction analysis (indicating taxa richness and diversity coverage). Relative abundance patterns of genera, families and phyla of baleen and toothed whale faecal microbiomes were compared by hierarchical clustering and non-metric multidimensional scaling (NMDS). Both analyses based on Bray-Curtis distances of the relative abundance patterns. For hierarchical clustering, the unweighted pair group method with arithmetic mean (UPGMA) method was used. One Way ANOSIM was performed to determine significant difference between community patterns of baleen and toothed whales. The analysis was based on Bray Curtis distances and 9999 permutations. Bonferroni-corrected *p*-values were used to indicate statistical significant differences among community patterns (*p* < 0.05). SIMPER analysis was used to calculate the contribution of each taxonomic group (given in % contribution) to the dissimilarity between each two groups, taxa that had the highest contribution to the differences among baleen and toothed whale microbiomes.

## Results

### Concentration of Bacterial and Archaeal 16S rRNA Gene Copies in Large Whale Faeces

**Fig. 1:**
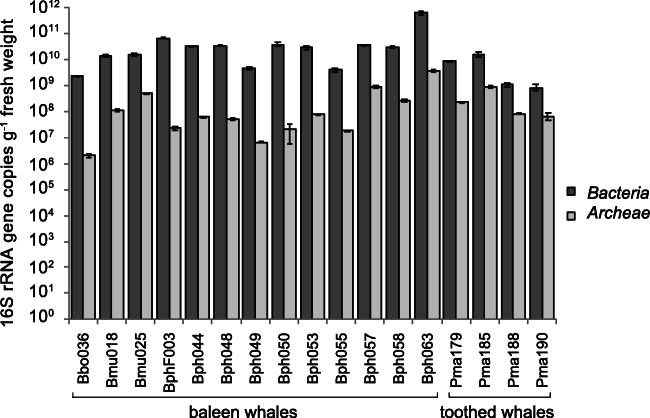
Concentrations of bacterial and archaeal 16S rRNA gene copies per g whale faeces. Analysis was performed by quantitative PCR using universal *Bacteria* and *Archaea* 16S rRNA gene targeting primer systems. Mean values and standard deviations of three technical replicates are given. FW: fresh weight

The concentration of bacterial and archaeal 16S rRNA gene copies in whale faecal samples was in the range of 10^9^ to 10^11^ and 10^6^ to 10^9^ copies per g fresh weight of faeces, respectively (Fig. 1; Supplementary Table S2). The concentration of bacterial 16S rRNA gene copies was always between one to four orders of magnitude higher than archaeal 16S RNA gene copies. No significant differences were obtained for the total bacterial and archaeal load in faecal samples among the whale families (*t*-tests; *Bacteria*: *p* = 0.18; *Archaea*; *p* = 0.74).

### Phylogenetic Community Profiling of the Bacterial Whale Microbiome - Illumina Sequencing Results

A total of 817,474 high-quality combined sequences were obtained from a total of 2,094,076 raw reads (raw read pairs 1,047,038) obtained by the amplicon sequencing of the 17 whale samples (Supplementary Table S3). Only 645 sequences (0.08% of the combined reads) were rejected for not meeting the quality criteria. The average length of the remaining sequences was 409 nucleotides (minimal length 52, maximal length 480 nucleotides). Based on a 98% sequence similarity value, a total number of 90,379 OTUs was obtained from 470,405 clustered sequences including 256,045 replicates. Finally, 816,829 sequences were classified, among those, 801,411 sequences (98.1% of the total sequences) were classified as *Bacteria* (9368 to 72,925 per sample), 38 as mitochondria (0.01%), 65 as chloroplasts (0.01%), 13,284 as *Archaea*, *Euryarchaeota* (1.6%), 232 as *Eukaryota* (0.03%) and 1799 sequences (0.2%) were summarised under “No Relative” (Supplementary Table S3).

Eukaryotic sequences were found in 11 of the 13 samples from the three baleen whale species, but in none of the four sperm whales*.* The sequences obtained were all identified as *Tetratrichomonas* (*Trichomonadea*, *Parabasalia*, *Metamonada*, *Excavata*).

### Relative Abundance of Different Bacterial Phyla in Large Whale Faecal Microbiomes

**Fig. 2:**
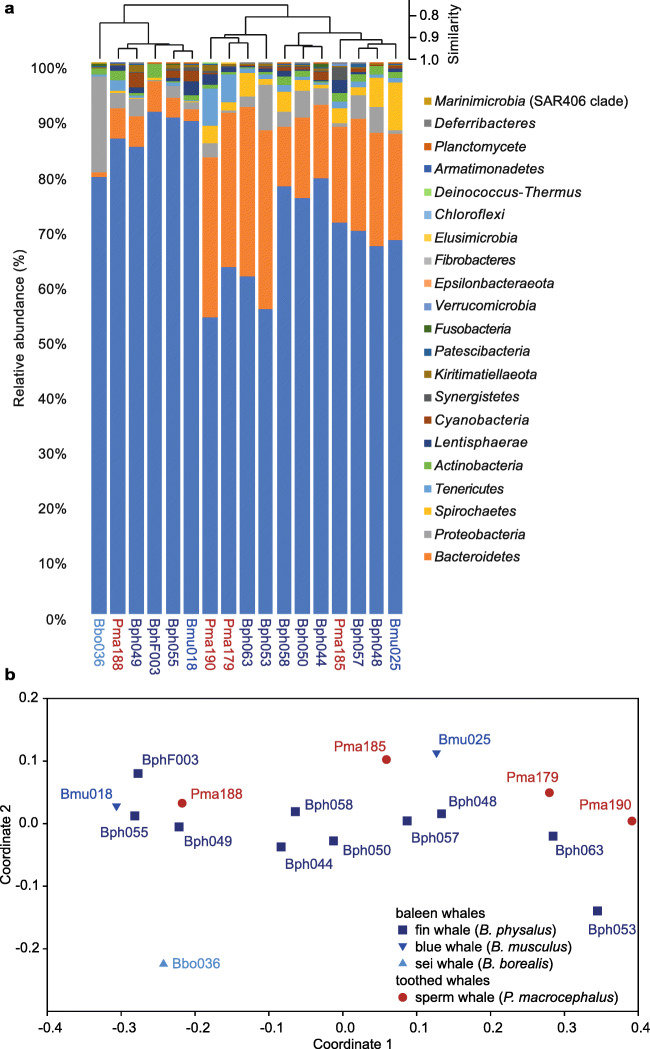
Differences in the composition of the baleen and toothed whale faecal microbiomes studied at the level of bacterial phyla. **a** Hierarchical clustering (UPGMA clustering based on Bray Curtis distances) and relative abundance heatmap. **b** NMDS plot based on Bray Curtis distances. All analyses were performed in PAST4

A total of 22 bacterial phyla were detected in the whale faecal samples (Fig. 2a; Supplementary Table S4), with nine phyla having a relative abundance above 1%. No distinct differences of the bacterial community patterns resolved at the level of phyla were visible by hierarchical clustering (Fig. 2a) and in the NMDS plot (Fig. 2b) between baleen and toothed whales. This was confirmed by One Way ANOSIM analysis which showed no significant differences in the phyla composition of baleen and toothed whale microbiomes (*p* = 0.2577).

*Firmicutes* was the predominant phylum in all cetacean faecal samples (53.9 to 91.2% of the sequenced 16S rRNA gene amplicons per sample; Fig. 2a; Supplementary Table S4). In 15 of the 17 samples, *Bacteroidetes* was the second most abundant phylum (0.9 to 32.4%). The two phyla together represented 80.1 to 96.7% of the bacterial 16S rRNA gene sequences obtained from the individual faecal samples. Changes in the relative abundance of *Firmicutes* and *Bacteroidetes* and the ratio among their relative abundance mainly affected the formation of different clusters in the hierarchical clustering based on relative abundance patterns of phyla (Fig. 2a, b).

The composition of the faecal microbiome at the level of bacterial phyla of the single sei whale (Bbo036) was clearly different to all other faecal microbiomes (Fig. 2). The microbiome of this sei whale sample showed a very high relative abundance of *Firmicutes* and a very low abundance of *Bacteroidetes* (79.2% and 0.9%; Fig. 2; Supplementary Table S4).

Beside the two dominating phyla, *Proteobacteria* followed by *Spirochaetae* and *Tenericutes* occurred at least in one of the samples with a relative abundance >6% and contributed with 8.1, 7.8 and 5.3%, respectively, to the differences among the individual faecal microbiomes (SIMPER analysis; Supplementary Tab. S4). *Proteobacteria* represented 0.2 to 4.9% of the bacterial 16S rRNA gene sequences in faecal microbiomes. Only in the sei whale sample (Bbo036) and in one fin whale sample (Bph053) the relative abundance of *Proteobacteria* was higher than that of *Firmicutes*, with 17.4% and 8.4%, respectively (Fig. 2a; Supplementary Tab. S4). The high abundance of *Proteobacteria* in those two samples was the main factor that led to the separation of the faecal microbiomes in the NMDS plot (Fig. 2b). The high abundance of *Proteobacteria* in the sei whale sample was outside the range of relative abundances of *Proteobacteria* obtained in the other microbiomes (Supplementary Table S4). The phylum *Spirochaetae* occurred in a relative abundance between 0.1 and 5.3%, and, except for one of the two blue whale samples (Bmu025), the relative abundance of *Spirochaetae* was with 8.7% above that range (Supplementary Table S4). The phylum *Tenericutes* occurred in a range of 0.1 to 1.8% and, except in two sperm whale samples (Pma179 and Pma190), the relative abundance was above the range (5.0% and 6.7%, respectively; Supplementary Table S4). The phyla *Lentisphaerae*, *Actinobacteria*, *Cyanobacteria*, *Synergistetes* and *Kiritimatiellaeota* occurred at least in one of the faecal microbiomes with a relative abundance of >1 to 2.5% and contributed with 1 to 2.7% to the differences among bacterial communities (Fig. 2a; Supplementary Table S4). Other detected but low abundant phyla (<1%) were *Fusobacteria*, *Verrucomicrobia*, *Epsilonbacteraeota*, *Fibrobacteres*, *Elusimicrobia*, *Chloroflexi*, *Deinococcus-Thermus*, *Armatimonadetes*, *Planctomycetes*, *Deferribacteres* and *Marinimicrobia* (SAR406 clade; Fig. 2a; Supplementary Table S4).

### Family Level Resolution Showed Distinct Bacterial Faecal Microbiomes of Baleen and Toothed Whales

**Fig. 3:**
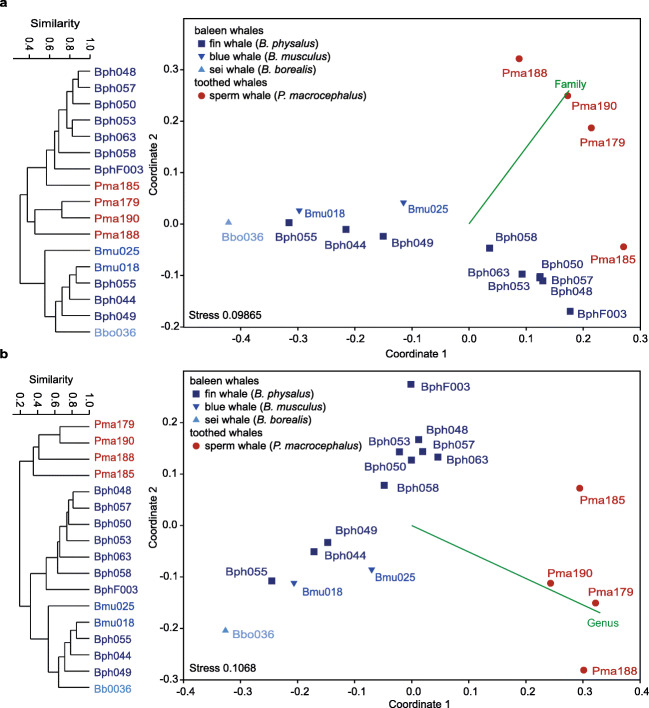
Differences in the composition of the baleen and toothed whale faecal microbiomes studied at the level of bacterial families (**a**) and genera (**b**). Analyses included hierarchical clustering based on Bray Curtis dissimilarity values and UPGMA clustering (left sides) and NMDS plots (right side) also using the Bray Curtis index for the generation of a dissimilarity matrix. Analysis was performed in PAST4

In total, 174 bacterial family level taxonomic paths were detected in the faecal microbiomes (Supplementary Table S5). Hierarchical clustering and the NMDS plots based on relative abundance patterns of family level taxonomic paths showed distinct non-overlapping patterns for baleen and toothed whale microbiomes (Fig. 3a). One Way ANOSIM confirmed that these differences were significant (*p* = 0.0041).

Different families of the orders *Clostridiales* (phylum *Firmicutes*) and *Bacteroidiales* (phylum *Bacteroidetes*) occurred with a high relative abundance either in baleen and toothed whale samples and had a high contribution to differences among the faecal microbiomes of the two groups of whales (SIMPER analysis; Supplementary Table S5; Supplementary Fig. 3). The families *Clostridiaceae* 1 (family taxon ID F-81) and *Bacteroidaceae* (F-27) occurred, respectively, with a 31.5- and 79-fold significant higher relative abundance in baleen than in toothed whales. In contrast, the *Clostridiales* vadinBB60 group (F-85) was present in considerable quantity in sperm whales (1.98–20.7%), but with only 0.03–1.41% in baleen whales. *Carnobacteriaceae* of the *Lactobacillales* (F-72; *Firmicutes*) were present in baleen whales with a relative abundance of 0.03 to 18.2% (mean 4.18 ± 5.7%), but not in sperm whales. The highest contribution to the differences of the bacterial microbiome composition (SIMPER analysis; Supplementary Table S5) was made with 22.11% by a *Clostridiales* 1 taxon (F-81). It occurred with a significantly higher relative abundance 82.75% [±26.3%]) in baleen than in toothed whales (0.87% [±0.89]). The second highest contributing family (14.35%) was *Ruminococcaceae* (F-95) of the order *Clostridiales* (*Firmicutes*). This family occurred with a mean relative abundance of 31.4% (±20.3%) in baleen and 28% (±13.3%) in toothed whales. An exception was the sei whale where the relative abundance of *Ruminococcaceae* was much lower (1.3%). Only few families occurred with a high relative abundance only in individual whale faeces microbiomes; *Pasteurellaceae* (F-156) and the *Clostridiales* family XIII (6.9%) occurred with a relative abundance of 15.9% and 6.9% only in the faecal sample of the sei whale and the *Synthrophomonadaceae* (F-96; *Clostridia*) and the *Bacteroidetes* BS11 group (F-28) with 11.0 and 7.9% only in one of the toothed whales (Pma185), respectively. The high relative abundance of F-96 and F-28 in the Pma185 faecal microbiome had also a high contribution to the differences of this toothed whale faecal microbiome compared to the other three toothed whales.

### Genus Level Resolution Showed Distinct Bacterial Faecal Microbiomes of Baleen and Toothed Whales

The composition of the bacterial faecal microbiomes compared at the level of bacterial genera showed distinct differences for baleen and toothed whale microbiomes by hierarchical clustering and in the NMDS plot (Fig. 3b). One Way ANOSIM analysis confirmed that the differences for baleen and toothed whale bacterial community patterns were statistically significant (*p* = 0.0003).

Slight but non-significant differences were found between the faecal microbiomes of the different *Balaenoptera* species, although a statistical comparison was not possible because of the small sample sizes. One fin whale (BphF003) faecal microbiome differed from those of the remaining fin whales.

### Distinct Abundant Genera Contributed to Baleen and Toothed Whale Faecal Microbiome Differences

**Fig. 4:**
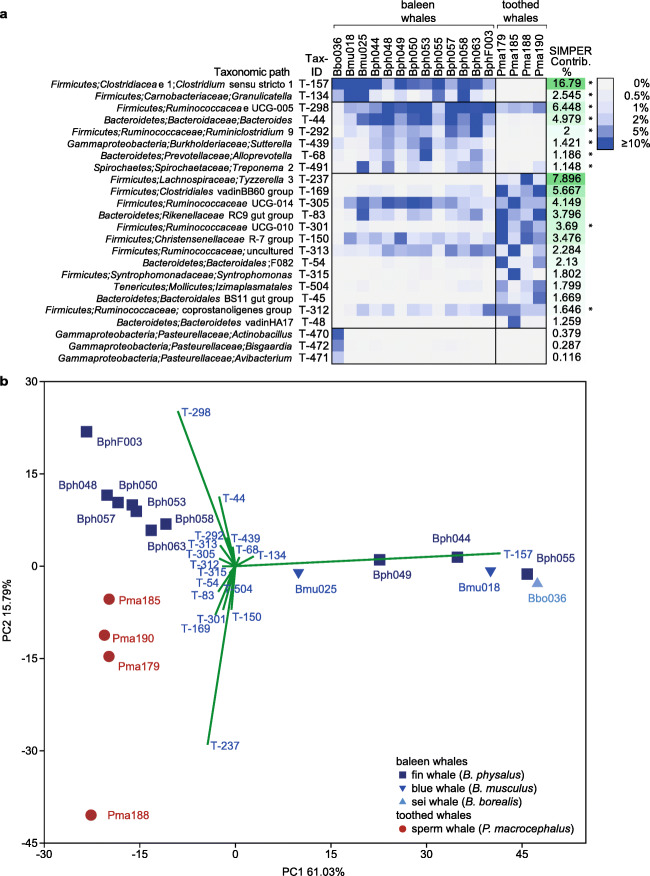
Impact of 21 most abundant genera (genus-like cluster) on differences among baleen and toothed whale faecal microbiomes. **a** Heatmap depicting relative abundances of the most abundant genera within the faecal microbiomes. Stars at the right side of the heatmap mark genera which showed significant differences in relative abundances in baleen and toothed whale faecal microbiomes (*t*-tests; *p* < =0.05). SIMPER values (calculated in PAST4) represent the contribution (in %) of individual phylogenetic groups on the differences of the baleen and toothed whale faecal microbiomes. A respective heatmap depicting all genera is given in Supplementary Table S6. **b** PCA plot depicting differences in the community profiles determined from the different whale species. The influence on individual genera on the community differences is depicted in form of biplots. Analysis was performed in PAST4 and based on relative abundance patterns of individual genera. Tax-IDs given at biplots are assigned to genera in **a**

In total 513 different genus level taxonomic paths have been identified. Among those, 58 occurred with a relative abundance of >1% in at least one faecal microbiome. Among those, the 21 most abundant contributed the most (>1%) differences among baleen and toothed whale faecal microbiomes as determined by SIMPER analysis (Supplementary Table S6; Fig. 4). *Firmicutes* (12 out of 21) followed by *Bacteroidetes* (6 out of 21) represented most of the abundant genera (Fig. 4a).

Eight of the genera occurred with a statistically significant higher relative abundance in baleen whales (*p* < 0.05; pairwise *t*-tests; Fig. 4a). The most abundant genus with the highest contribution to community differences (SIMPER 17.0%; Fig. 4a) was the genus-like cluster *Clostridium sensu stricto* 1 (T-157) of the *Clostridiaceae* (F-81). Sequences assigned to this cluster occurred with a high relative abundance of up to 66% in the faecal samples of the different *Balaenoptera* species (Fig. 4). The taxon occurred with a very high relative abundance (29–56%; mean 53%) in six out of 13 *Balaenoptera* whales including one sei whale*,* two blue whales and three fin whales. The taxon mainly contributed to the distinction of those microbiomes from other microbiomes as shown by the box plot analysis in the PCA plot (Fig. 4b). The relative abundance of this taxon in the other fin whale faecal microbiomes was between 2.6 and 9.1% (mean 5.6%). The taxon was only detected with a very low relative abundance (0.1%) in one of the four sperm whale faecal microbiomes (Fig. 4a). A second *Firmicutes* genus (*Granulicatella*, T134) had the same occurrence pattern as T-157 and had with 2.6% a countable contribution to the differences of the baleen and toothed whale faecal microbiomes. The remaining six genera with a significantly higher relative abundance in baleen whales and high contribution to the microbiome differences were two uncultured *Firmicutes* genus-like cluster, *Ruminococcaceae* UCG-005 (T298) and *Ruminiclostridium* 9 (T292), two *Bacteroidetes* genera, *Bacteroides* (T-44) and *Alloprevotella* (T-68) and the genera *Sutterella* (T-439) of the *Burkholderiaceae* (*Gammaproteobacteria*) and *Treponema* 2 (T-49) of the *Spirochaetaceae* (*Spirochaetes*).

Several of the above-mentioned genera were less abundant in the faecal microbiome of the studied sei whale. The sei whale faecal microbiome contained, in contrast, a high relative abundance of three *Pasteurellaceae* genera, i.e. *Actinobacillus* (T470; 8.0%), *Bisgaardia* (T-472; 5.4%) and *Avibacterium* (T-471; 2.4%). The genera occurred only with a total of 0.8% in faecal samples of the other baleen whales and were not detected in the four sperm whales.

Conversely to the baleen whales, 12 genera occurred with a high relative abundance in toothed whale faecal microbiomes and had a high contribution to detected microbiome differences. The most abundant genera with high contribution to microbiome differences (SIMPER value >5%, Fig. 4a) were two *Firmicutes* genus-like cluster, *Tyzzerella* 3 (T-237; *Lachnospiraceae*) and the *Clostridiales* vadinBB60 group (T-169). Six additional *Firmicutes* genera, including three uncultured *Ruminococcaceae* genus-like cluster (T-305, T-301, T-312), the uncultured *Christensenellaceae* genus-like cluster R-7 (T-150) and the genus *Synthrophomonas* (T-315), three uncultured *Bacteroidetes* genus-like cluster assigned to the *Rikenellaceae* (T-83) and *Bacteroidales* (T-54, T-48) and one *Tenericutes* genus-like cluster (T-504; *Izimaplasmatales* of the *Mollicutes*), occurred with a higher relative abundance in toothed whale faecal microbiomes and contributed with 1.3 to 4.1% (SIMPER values; Fig. 4a) to the differences among the baleen and toothed whale faecal microbiomes.

### Baleen and Toothed Whale Faecal Microbiomes Showed No Distinct Differences with Respect to Alpha Diversity Parameters

**Fig. 5:**
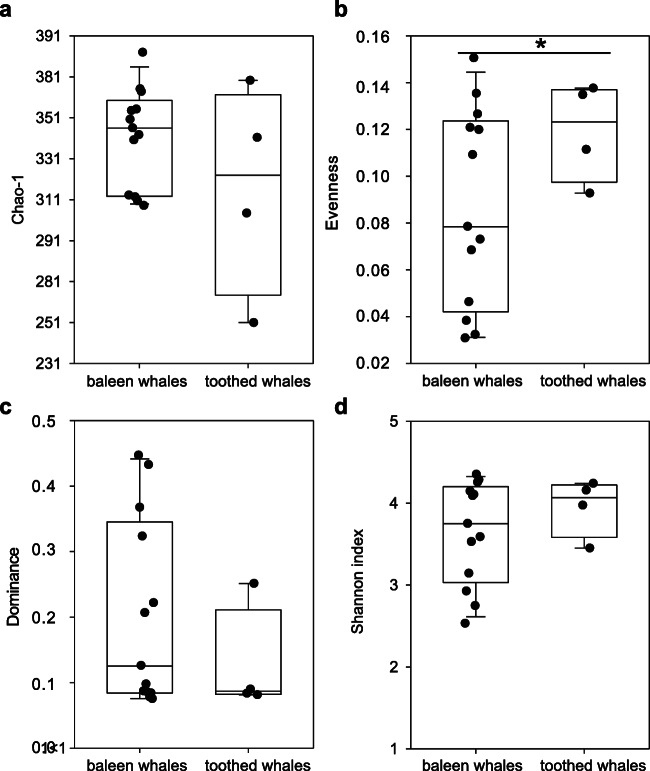
Comparison of baleen and toothed whale faecal microbiome at the genus level by alpha diversity parameters. **a** Richness (Chao-1). **b** Eveness. **c** Dominance. **d** Shannon index. Values obtained for baleen and toothed whales microbiomes were compared by *t*-tests. Significant differences (*p* < = 0.05) are marked with a star at top of the box-plots

The alpha diversity values including the number of detected genera/genus-like cluster (Chao-1 index; Fig. 5a) and the distribution of those genera within the microbiomes (evenness, dominance values) and the overall diversity (Shannon diversity index; Fig. 5b–d) showed some variations among individual faecal microbiomes but without distinct patterns among baleen and toothed whales. Significant differences in genera distributions between baleen and toothed whale faecal microbiomes were only determined for the evenness (*t*-test; *p* < 0.05; Fig. 5d; Supplementary Table S7). Especially four of baleen whale faecal microbiomes were characterised by low evenness values combined with high dominance values indicating a high relative abundance of few bacterial genera (Bbo036, Bmu018, Bph044, Bph055; Fig. 5; Supplementary Table S7). Rarefaction curves for all faecal microbiomes indicated that the complete diversity at the genus level was not yet covered with the total number of analysed sequences per sample (Supplementary Fig. S4). However, the investigation based on the dataset of faecal microbiomes gave a clear overview of most abundant genera and indicated clear differences between faecal microbiomes of blue, sei, fin and sperm whales.

### Low Abundance of Archaeal 16S rRNA Gene Sequences in Large Whale Microbiomes

**Fig. 6:**
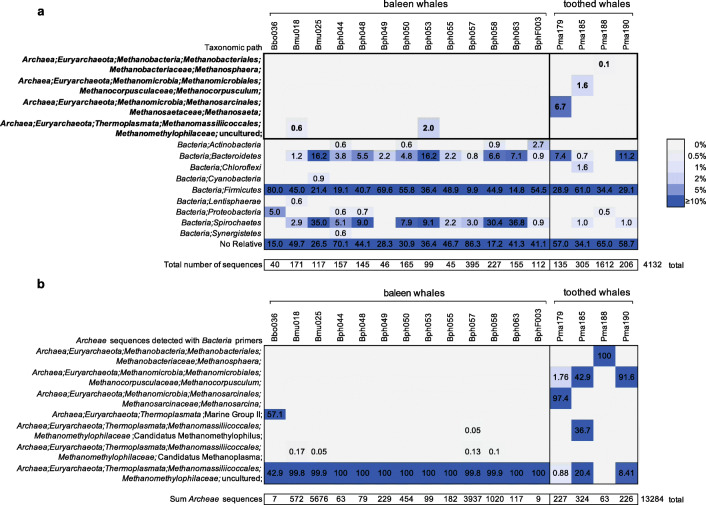
Relative abundances and absolute numbers of archaeal 16S rRNA gene sequences detected by the 16S rRNA gene Illumina amplicon sequencing of baleen and toothed whale faecal microbiome using archaeal (**a**) and bacterial (**b**) specific primer systems

Because qPCR analysis indicated the presence of *Archaea* in all 17 large whale faecal samples, archaeal 16S rRNA gene amplicon libraries were generated as well as using a recommended archaeal-specific primer system [26]. In summary, only 70,038 combined reads could be subjected to phylogenetic analysis. Among these, 65,906 reads (94.1%) of the sequences were rejected because they did not match the quality criteria of the SILVAngs analysis pipeline. The average length of the finally aligned sequences was 173 nt (35–493 nt). A total number of 689 OTUs (1.0%) was obtained. In total, 456 sequences were clustered (0.65%) and 2987 reads counted as replicates (4.68%). Only 1875 sequences were classified (2.68%) and 2257 sequences were summarised under the criterion of “No Relative” (3.22%). From the 1875 classified sequences, only 19 sequences represented *Archaea*; the remaining sequences were identified as *Bacteria*, mainly of the *Bacteroidetes* and *Firmicutes* as well as some *Spirochaetes*, *Alpha-* and *Gammaproteobacteria* and *Lentisphaerae* (Fig. 6a; Supplementary Table S8)

More archaeal sequences were determined with the *Bacteria* 16S rRNA gene targeting primer system (13,284 sequences; Fig. 6b). All detected *Archaea* sequences represented *Euryarchaeota*. The most abundant archaeal taxon in baleen whales was a genus level cluster of uncultured *Methanomethylophilaceae* (class *Thermoplasmata*; 42.9-100% of the archaeal sequences per samples). This taxon was less abundant in sperm whales (0–20% of archaeal sequences). Two additional genera of *Thermoplasmata* occurred in high relative abundances in the sei whale (Bbo036; Marine Group II; 57.1% of the archaeal sequences) and in one of the sperm whales (Pma185; *Methanosarcina*, 36.7%). Further archaeal sequences were assigned to the class *Methanobacteria.* These sequences were assigned to the genera *Methanosphaera* (class *Methanobacteria*) and *Methanocorpusculum* (class *Methanomicrobia*) and *Methanosarcina* (class *Methanomicrobia*).

## Discussion

### Specificity of the Research on the Intestinal Microbiomes of Free-Ranging Large Whales

Research on intestinal microbiomes of free-ranging, presumably healthy large whales is scarce. A key problem is the specificity of the sampling technique. Due to the challenges in sampling free-swimming cetaceans, currently only faecal samples can be used to get information on the gut microbiome of live animals in the wild. The available information on the microbiome composition for different regions of the gastrointestinal (GI) tract is limited to few studies [19, 34]. For example, Wan et al. [34] studied the stomach, foregut and hindgut in comparison to the faeces microbiomes of five East Asian finless porpoises immediately after their death due to by-catch. The study showed that the richness of the different gut microbiomes was even, but the phylogenetic composition was different between hindgut and faeces to stomach and foregut [34]. In conclusion, the faecal microbiome studied here is representative for the large intestine (i.e. colon, caecum, rectum); differences of microbiomes at more proximally located intestinal segments cannot be ruled out.

Another problem created by sampling floating faeces is that it cannot be excluded that bacterioplankton of the surrounding water may be present in the sequenced microbiome. No seawater analysis has been conducted here, but earlier studies have already shown that marine mammal microbiomes are different from those of, e.g., dietary fish and seawater [1]. This was also confirmed by Apprill et al. [35] for the skin microbiome of humpback whales (*Megaptera novaeangliae*).

### Methanogenic *Archaea* in the Faecal Microbiomes of Large Whales

The present study is one of the first to specifically attempt to analyse also the *Archaea* composition besides that of *Bacteria* in the faecal microbiome of large whales. Archaeal sequences were found in all faecal samples by quantitative PCR using a common archaeal 16S rRNA gene targeting primer systems, but with a low concentration of approximately two log scores compared to *Bacteria*. The vast minority of these sequences may have been archaeal 16S rRNA gene sequence targets as determined by the archaeal 16S rRNA gene amplicon sequencing. The detected archaeal sequences were primarily assigned to the order *Methanomassiliicoccales* representing potential methanogenic *Archaea*. So far it is known that habitat-specific clades of *Methanomassiliicoccales* occur in the intestinal tracts of different animals [36] and the composition of *Methanomassiliicoccales* can vary with different diets as shown for example in a study of caprine intestine [37]. Other archaeal sequences detected here, *Methanosphaera* (class *Methanobacteria*) and *Methanocorpusculum* and *Methanosarcina* (both *Methanomicrobia*), also represented different methanogens which differed in their metabolic pathways to produce methane [38–40].

### Abundance of mitochondriate Unicellular Protists

The *Bacteria* 16S rRNA gene amplicon approach gave also some information on mitochondriated intestinal flagellated unicellular protists with parasitical lifestyle: *Tetratrichomonas* (*Trichomonadea*, *Parabasalia*, *Metamonada*, *Excavata*). Trichomonads in general are found in the intestine and urogenital tract as common parasites of many vertebrate and invertebrate species [41–43]. In previous studies on occurrence of gastrointestinal parasites of sperm [3], blue [44–46], sei and fin whales [44, 45], the genus *Tetratrichomonas* or any other closely related genera were not detected as whale parasites [44].

### Comparison of Bacterial Microbiome Data Available for Large Whales and Other Marine Mammals

A high abundance of *Bacteroidetes* and *Firmicutes* has been found in other whale microbiomes and also in other terrestrial mammals [14]*.* However, *Bacteroidetes* were generally more abundant than *Firmicutes*. Studies of the faecal microbiomes of humans and mice and several other terrestrial mammals showed a relative abundance of 60–80% *Bacteroidetes* and 20–40% *Firmicutes* [12]. Dominant roles for these two phyla were found in faecal microbiomes of different baleen whale species [14], two live blue whales [18], stranded pygmy and dwarf sperm whales [16], one sperm whale [47] and southern right whale calves [17]. The faecal samples of the two blue whales analysed by Guass et al. [18] were dominated by *Firmicutes* (relative abundance >98%); in the study of Sanders et al. [14] some faecal microbiomes were also dominated by *Firmicutes* while others showed a higher abundance of *Bacteroidetes* followed by *Firmicutes*. In contrast to our study, the taxonomic composition of the *Odontoceti* faecal microbiomes investigated by Sanders et al. [14] strongly varied among the samples and was enriched in *Proteobacteria* and *Fusobacteria*. In the present study, the microbiome composition was quite stable among the four sperm whale samples (analysed at the level of genera) and *Proteobacteria* were detected only in low abundances (0.4 to 2.6%). *Fusobacteria* were only detected in a single sperm whale sample and in very low abundance (0.004%). These contrasting results may be due to differences in intestinal microbiome related to feeding ecology within the *Odontoceti.* All of the *Odontoceti* samples in our study belong to sperm whales, while in Sanders et al. [14], *Odontoceti* samples originated from three different dolphin species. In temperate latitudes, such as in the Azores, sperm whales are chiefly teuthophagous, although they may occasionally consume small amounts of fish [48, 49]. Dolphins, on the other hand, have a more generalist diet. For example, the Atlantic white-sided dolphin (*Lagenorhynchus acutus*), the bottlenose dolphin (*Tursiops truncatus*) and the beluga whale that were investigated by Sanders et al. [14] feed on coastal and pelagic fish and cephalopods (and other invertebrates in the case of the beluga), and their diet can vary seasonally and geographically [50–52]. Furthermore, aside from one Atlantic white-sided dolphin, samples from *Odontoceti* in the study of Sanders et al. [14] were all from captive dolphins; thus, the results may not be completely representative of wild populations.

On the other hand, our study is in agreement with the findings of Sanders et al. [14] regarding a relatively high abundance of *Spirochaetae* in the faecal microbiome of baleen whales. This was pointed out as a clear difference to the low abundance of *Spirochaetae* in faecal microbiome of terrestrial mammals (Sanders et al. [14]). In line with our results, Sanders et al. [14] also detected a very low proportion of *Proteobacteria* in baleen whale samples. Within this phylum, *Pasteurellaceae* were exclusively found in the sei whale in significant numbers (15.9%), and this group was also detected with a relative abundance of 4 ± 1.9% in a 16S rRNA gene–based Illumina amplicon sequencing study of the faecal microbiome of adult dolphins [15]. The ecological niche of *Bisgaardia* spp. is the oropharynx of seals and sea elephants, but the other two members of *Pasteurellaceae*, *Actinobacillus* and *Avibacterium*, also contain species colonising terrestrial mammals and birds, respectively.

Although cetaceans evolved from herbivorous land-living artiodactyls and still share a number of “ruminant-like” anatomical homologies to this group, large baleen whale microbiota composition parallels that of both terrestrial herbivores and carnivores, especially with respect to functional capacity (chitin degradation) and taxonomic level [14]. Sperm whale microbiomes determined in this study differed in several aspects from that of baleen whales. Specifically, differences were found for the relative abundance of different *Firmicutes* and *Bacteroidetes* taxa which were either present in the baleen or toothed whale faecal samples. Among those were several taxa of uncultured *Ruminococcaceae* and uncultured clostridia, which may have specific nutritional functions in the intestinal microbiota of sperm and baleen whales. This suggests that their marine diet compositions have significantly influenced co-evolution of commensal intestinal microbes in these two whale families.

The sei whale faecal microbiome analysed in our study showed strong differences to the other two baleen whale species, which can be explained by different diet and spatial distribution. The chief difference that was found was the high abundance of *Pasteurellaceae* (*Proteobacteria*). Another sei whale microbiome, inferred from a sample collected in 2011 off the Canadian Atlantic coast, revealed a much lower abundance of *Proteobacteria*, which may indicate spatiotemporal or individual differences and warrant further investigation into these aspects [14, 16].

*Tenericutes* and *Proteobacteria* were, together with *Bacteroidetes*, determined by 454 pyrosequencing among the most abundant phyla in the foregut of dolphins [1]*.* In contrast, *Firmicutes* was among the most abundant phyla detected in the rectal samples of dolphins together with *Proteobacteria* and *Fusobacteria* while *Bacteroidetes* occurred only in a very low abundance in those samples (<1%; [1]). *Tenericutes* and *Proteobacteria* were also quite abundant in faecal microbiomes in this study, while *Fusobacteria* occurred in a very low relative abundance in all whale faecal samples (<0.8%). Another study of adult dolphins determined *Firmicutes* and *Proteobacteria* as the most dominating phyla in the faecal microbiome [15]. In that study, *Actinobacteria*, *Bacteroidetes*, *Fusobacteria* and *Tenericutes* were determined together (each of them in a relative abundance of 3 to 5%) and thus were accounted as subdominant phyla in respective faecal samples. Studies of faecal microbiomes of wild young South American and subarctic fur seals as well as Australian sea lions also revealed that *Firmicutes* represented the most abundant phylum with more than 80% in wild marine mammals [53, 54]. In contrast to our study, the relative abundance of *Bacteroidetes* was in general very low. *Fusobacteria* occurred with a very low relative abundance. The distribution of *Actinobacteria* and *Proteobacteria* varied between the four seal species and was higher than that found in the whale samples. Besides our faecal whale microbiome study, a skin microbiome study (epimicrobiome study) of 19 North Pacific humpback whales revealed also a high proportion of *Bacteroidetes* [35].

The alpha diversity of the faecal bacterial microbiomes of the baleen and toothed whales showed no statistically significant differences except for four of the baleen whales’ faecal microbiomes, which were characterised by low evenness combined with high dominance values indicating a high relative abundance of few bacterial genera (Bbo036, Bmu018, Bph044, Bph055). Erwin et al. [16] also determined no significant differences in alpha diversity for intestinal microbial communities of kogiid hosts. Diversity and faecal microbiome composition of pygmy and dwarf sperm whales were recently found to be significantly more diverse (mean 416–432 OTUs) compared to other toothed whales [14, 16]. In contrast to rectal microbiomes of wild and captive dolphins, porpoises and beluga whales that contained 10–179 OTUs on average [1, 34, 47, 55] with 122 a higher number than in this study was noted in a single sperm whale faecal microbiome [47]. A mean number of 64–87 OTUs was found in the present study. Higher values of 163–364 OTUs on average were also reported in baleen whales [47] compared to 56–77 OTUs from this study.

Interestingly, the low level of diversity in intestinal and faecal microbiomes was also found in this study contrasts with other body sites of cetaceans, where a rich diversity of microbial communities has been documented. Bik et al. [1] detected twice the phylum level and four times the OTU level diversity in oral microbiomes of bottlenose dolphins compared to their intestinal microbiomes.

### Detection of Potential Pathogens in the Bacterial Whale Microbiomes

Although the whales from this study were apparently healthy, the dataset was checked for known and potential bacterial pathogens in cetaceans and other marine mammals, in accordance with the One Health principle [56]. In these, members of genera *Staphylococcus*, *Streptococcus*, *Erysipelothrix*, *Clostridium*, *Mycobacterium*, *Chlamydia*, *Brucella*, *Leptospira*, *Aeromonas*, *Pseudomonas*, *Plesiomonas*, *Edwardsiella*, *Salmonella*, *Campylobacter* and *Helicobacter* were considered animal pathogens, sometimes also associated with human infections [17, 55, 57–66]. From the latter, very similar bacteria to the rat bite fever organism, *Streptobacillus moniliformis*, were recently described as *Oceanivirga* in fish, dolphins, sea lions and different cetacean species [1, 67–69]. In this regard, it needs to be explicitly mentioned that amplicon studies targeting partial 16S rRNA gene sequences alone cannot resolve taxa reliably at species level [21]. Sequences, assigned to the genera *Chlamydia*, *Brucella*, *Leptospira*, *Aeromonas*, *Pseudomonas*, *Plesiomonas*, *Edwardsiella*, *Salmonella* and *Oceanivirga* (*Leptotrichiaceae*) were not detected. Sequences of the following genera were detected in our dataset, although the resolution was not high enough to unequivocally identify certain taxa to species level (in brackets putative pathogens and possibly zoonotic microorganisms): *Staphylococcus* (*St. aureus*), *Streptococcus* (*S. phocae*, *S. iniae*, *S. suis*, *S. pyogenes*, *S. canis*, *S. zooepidemicus*), *Erysipelothrix* (*E. rhusiopathiae*), *Clostridium* (*C. perfringens*), *Mycobacterium* (*M. pinnipedii*, *M. abscessus*), *Campylobacter* (*C. fetus*) and *Helicobacter* (*H. pylori*, *H. cetorum*)*.*

## Conclusions

Albeit remarkable differences exist between large baleen and toothed whale families specifically with respect to feeding ecology, there was a high degree of homology in the microbiomes in both groups. In this study, differences were detected especially at the lower taxonomic levels (genera/taxonomic groups), that can help to address different pathways in food digestion. However, even the scarce number of similar studies has shown that striking differences remain within and between species, as well as between different biomes, questioning a strict host-microbe specificity and suggesting important influences of season, diet and even social contacts, as described earlier [55, 70]. Our results warrant further investigation on the role of these effects in shaping cetacean microbiomes. Highly abundant phyla in most faecal samples were *Firmicutes*, followed by *Bacteroidetes*, *Proteobacteria*, *Tenericutes* and *Spirochaeta* occurred in individual samples with a relative abundance of up to 17.4%. Lower abundances were found for *Lentisphaerae*, *Cyanobacteria*, *Actinobacteria*, *Synergistetes*, *Verrucomicrobia* and *Fusobacteria* in some of the samples. *Archaea* were found in the minority of samples and with a low number of different taxa, all known as methanogens. A relatively small number of potentially pathogenic and even zoonotic species were detected. Further studies are needed to better understand these differences, especially taking into account the poor conservation status of many cetacean species and populations worldwide. So far, most of the data available are descriptive and little is known about functions of the different taxa in the intestinal microbiota of marine mammals including whales.

## Supplementary Information

ESM 1 (DOCX 28824 kb).

## Data Availability

Raw data of paired-end sequence read of bacterial and archaeal 16S rRNA gene amplicon sequences are available at the Sequence Read Archive (SRA) under BioSample accessions number SRR11586677- SRR11586693 within the BioProject PRJNA627228.

## Code Availability

All details to applied software including version numbers are given in the text.
