# Supplementary material for: A Preliminary Comparison on Faecal Microbiomes of Free-Ranging Large Baleen (Balaenoptera musculus, B. physalus, B. borealis) and Toothed (Physeter macrocephalus) Whales
Source: Microb Ecol. 2021 Mar 21;83(1):18–33. doi: 10.1007/s00248-021-01729-4 (PMC8881428; doi:10.1007/s00248-021-01729-4)
Supplement: Supplementary file 1 — (DOCX 28824 kb). [file 248_2021_1729_MOESM1_ESM.docx]

**Supplementary Data**

**A preliminary comparison on faecal microbiomes of free-ranging large baleen (*Balaenoptera musculus*, *B. physalus*, *B. borealis*) and toothed (*Physeter macrocephalus*) whales**

# Stefanie P. Glaeser^1,*^, Liliana M. R. Silva^2^, Rui Prieto^3,4^, Mónica A. Silva^3^, Angel Franco^1^, Peter Kämpfer^1^, Carlos Hermosilla^2^, Anja Taubert^2^, Tobias Eisenberg^5^

^1^Institute of Applied Microbiology, Justus Liebig University Giessen, Giessen, Germany

^2^Institute of Parasitology, Justus Liebig University Giessen, Germany

^3^ Institute of Marine Research (IMAR) and Okeanos R&D Centre, University of the Azores, Horta, Portugal

^4^ MARE—Marine and Environmental Sciences Centre, Lisbon, Portugal

^5^ Hessian State Laboratory (LHL), Department of Veterinary Medicine, Giessen, Germany

*Corresponding author: Institute of Applied Microbiology, Justus Liebig University Giessen, Heinrich-Buff-Ring 26-32, 35392 Giessen, Germany.

*E-mail*: Stefanie.Glaeser@umwelt.uni-giessen.de

**Supplementary Tables**

**Supplementary Table S1** Overview of investigated whale samples including information of whale species, geographical sampling locations, and sampling dates.

| **Ref** | **Species** | **Date** | **Long** | **Lat** | **Area** |
| --- | --- | --- | --- | --- | --- |
| Bbo036 | *Balaenoptera borealis* | 29/04/2014 | -28.4363 | 38.68585 | N Faial |
| Bmu018 | *Balaenoptera musculus* | 14/04/2014 | -28.54793 | 38.27137 | S Faial |
| Bmu025 | *Balaenoptera musculus* | 30/05/2014 | -28.34484 | 38.21362 | S Pico |
| BphF003 | *Balaenoptera physalus* | 23/05/2011 | -28.44594 | 38.25779 | S Pico |
| Bph044 | *Balaenoptera physalus* | 23/05/2014 | -28.33653 | 38.37943 | S Pico |
| Bph048 | *Balaenoptera physalus* | 28/05/2014 | -28.50404 | 38.30350 | S Pico |
| Bph049 | *Balaenoptera physalus* | 30/05/2014 | -28.45452 | 38.32296 | S Pico |
| Bph050 | *Balaenoptera physalus* | 30/05/2014 | -28.46797 | 38.30947 | S Pico |
| Bph053 | *Balaenoptera physalus* | 02/06/2014 | -28.11554 | 38.23060 | S Pico |
| Bph055 | *Balaenoptera physalus* | 19/06/2014 | -28.63609 | 38.37399 | S Faial |
| Bph057 | *Balaenoptera physalus* | 20/06/2014 | -28.38053 | 38.26626 | S Pico |
| Bph058 | *Balaenoptera physalus* | 20/06/2014 | -28.32310 | 38.24674 | S Pico |
| Bph063 | *Balaenoptera physalus* | 01/07/2014 | -28.63795 | 38.36766 | S Faial |
| Pma179 | *Physeter macrocephalus* | 08/07/2014 | -28.40947 | 38.24109 | S Pico |
| Pma185 | *Physeter macrocephalus* | 11/08/2014 | -28.30696 | 38.38137 | S Pico |
| Pma188 | *Physeter macrocephalus* | 22/08/2014 | -28.85874 | 38.24667 | S Faial |
| Pma190 | *Physeter macrocephalus* | 22/08/2014 | -28.88048 | 38.23622 | S Faial |

**Supplementary Table S2** Concentrations of bacterial and archaeal 16S rRNA gene targets in the faecal samples. Concentrations are given as gene targets per g fresh weight of feces. Mean values and standard errors of triplicate qPCR measurements are given.

| Samples^1^ | *Bacteria* | *Archaea* |
| --- | --- | --- |
| Bbo036 | 2.4 (± 0.03) × 10^9^ | 2.1 (± 0.4) × 10^6^ |
| Bmu018 | 1.3 (± 0.2) × 10^10^ | 1.1 (± 0.1) × 10^8^ |
| Bmu025 | 1.6 (± 0.2) × 10^10^ | 4.9 (± 0.2) × 10^8^ |
| BphF003 | 6.8 (± 0.2) × 10^10^ | 2.4 (± 0.4) × 10^7^ |
| Bph044 | 3.2 (± 0.1) × 10^10^ | 6.3 (± 0.1) × 10^7^ |
| Bph048 | 3.4 (± 0.2) × 10^10^ | 5.1 (± 0.7) × 10^7^ |
| Bph049 | 4.6 (± 0.4) × 10^9^ | 6.4 (± 0.4) × 10^6^ |
| Bph050 | 3.9 (± 0.6) × 10^10^ | 2.0 (± 0.1) × 10^7^ |
| Bph053 | 2.9 (± 0.3) × 10^10^ | 7.6 (± 0.4) × 10^7^ |
| Bph055 | 4.1 (± 0.6) × 10^9^ | 1.9 (± 0.04) × 10^7^ |
| Bph057 | 3.6 (± 0.2) × 10^10^ | 9.0 (± 0.6) × 10^8^ |
| Bph058 | 3.0 (± 0.4) × 10^10^ | 2.7 (± 0.4) × 10^8^ |
| Bph063 | 6.3 (± 1.1) × 10^11^ | 3.8 (± 0.4) × 10^9^ |
| Pma179 | 9.2 (± 0.2) × 10^9^ | 2.4 (± 0.01) × 10^8^ |
| Pma185 | 1.6 (± 0.4) × 10^10^ | 9.2 (± 1.0) × 10^8^ |
| Pma188 | 1.1 (± 0.2) × 10^9^ | 8.3 (± 0.3) × 10^7^ |
| Pma190 | 8.4 (± 0.2) × 10^8^ | 6.5 (± 2.1) × 10^7^ |

^1^ Sample abbreviations are depicted in Supplementary Table S1

**Supplementary Table S3** Overview of the number of combined sequences bacterial 16S rRNA gene amplicon sequences generated with the Illumina technology.

| **Samples** | **Raw total reads^1^** | **Raw read pairs^2^** | **Combined sequence reads^3^** | **Quality controlled classified combined sequences^4^** | **Bacteria** | **Chloroplast** | **Mito-chondria** | **Eukaryota; Excavata** | **Archaea^5^** | **No Relative** |
| --- | --- | --- | --- | --- | --- | --- | --- | --- | --- | --- |
| Bb0036 | 107,164 | 53,582 | 42,196 | 42,148 | 42,048 | 15 | 0 | 1 | 7 | 77 |
| Bmu018 | 163,286 | 81,643 | 64,221 | 64,179 | 63,541 | 8 | 6 | 8 | 572 | 44 |
| Bmu025 | 146,388 | 73,194 | 57,647 | 57,626 | 51,829 | 3 | 4 | 2 | 5,676 | 112 |
| Bph044 | 84,300 | 42,150 | 33,939 | 33,919 | 33,770 | 1 | 0 | 24 | 63 | 61 |
| Bph048 | 140,126 | 70,063 | 54,519 | 54,482 | 54,295 | 2 | 3 | 1 | 79 | 102 |
| Bph049 | 144,670 | 72,335 | 56,351 | 56,259 | 55,984 | 8 | 3 | 20 | 229 | 15 |
| Bph050 | 183,922 | 91,961 | 73,563 | 73,528 | 72,925 | 2 | 2 | 9 | 454 | 136 |
| Bph053 | 47,526 | 23,763 | 18,738 | 18,713 | 18,574 | 0 | 0 | 23 | 99 | 17 |
| Bph055 | 163,818 | 81,909 | 64,077 | 63,983 | 63,669 | 0 | 1 | 104 | 182 | 27 |
| Bph057 | 151,684 | 75,842 | 60,103 | 60,072 | 55,969 | 0 | 5 | 24 | 3,937 | 137 |
| Bph058 | 160,156 | 80,078 | 59,706 | 59,668 | 58,578 | 2 | 2 | 16 | 1,020 | 50 |
| Bph063 | 89,314 | 44,657 | 35,509 | 35,501 | 35,203 | 0 | 2 | 0 | 117 | 179 |
| BphF003 | 179,654 | 89,827 | 72,045 | 72,015 | 71,967 | 1 | 0 | 0 | 9 | 38 |
| Pma179 | 96,836 | 48,418 | 35,116 | 35,098 | 34,699 | 3 | 0 | 0 | 227 | 169 |
| Pma185 | 152,616 | 76,308 | 57,332 | 57,239 | 56,643 | 2 | 0 | 0 | 324 | 270 |
| Pma188 | 23,730 | 11,865 | 9,457 | 9,453 | 9,368 | 7 | 6 | 0 | 63 | 9 |
| Pma190 | 58,886 | 29,443 | 22,955 | 22,946 | 22,349 | 11 | 4 | 0 | 226 | 356 |
| Total | 2,094,076 | 1,047,038 | 817,474 | 816,829 | 801,411 | 65 | 38 | 232 | 13,284 | 1,799 |
| Total (%) | - | - |  | 100 | 98.11 | 0.01 | 0.00 | 0.03 | 1.63 | 0.22 |

^1^ Raw total reads obtained by Illumina amplicon sequencing, the number was reduced by further sequence processing including adaptor and primer sequence clipping as well as combining of paired end reads.

^2^ Raw read pairs, the number was reduced by further sequence processing including adaptor and primer sequence clipping as well as combining of the paired end reads.

^3^ Total number of combined sequences before Silva NGS analysis.

^4^ Total number of combined sequences which passed the quality control in the SilvaNGS analysis.

^5^ *Eukaryota, Excavata, Metamonada, Parabasalia, Trichomonadea, Tetratrichomonas*

**Supplementary Table S4** Relative abundance of bacterial phyla in whale faecal samples. Depicted values represent relative abundances given in percent (%). Samples are sorted according to whale genera and species. Most abundant phyla are given at the top. Mean and standard deviations (SD) as relative abundances for the two whale families. Heatmap colors indicate relative abundance. SIMPER analysis indicates the contribution of individual phyla on differences among the composition of the faecal microbiomes of the investigated baleen and toothed whales. T-tests indicate if differences in relative abundance of individual phyla in baleen and toothed whales were significant (p<0.05).

**Supplementary Table S5** Relative abundance of bacterial families in whale faecal samples. Depicted values represent relative abundances given in percent (%). Samples are sorted according to whale genera and species. Most abundant families are given at the top. Mean and standard deviations (SD) as relative abundances for the two whale families. Each detected family was assigned with an unique family tax-ID (F-number). Heatmap colors indicate relative abundance. SIMPER analysis indicates the contribution of individual phyla on differences among the composition of the faecal microbiomes of the investigated baleen and toothed whales. T-tests indicate if differences in relative abundance of individual phyla in baleen and toothed whales were significant (p<0.05).

**Supplementary Table S6** Relative abundance of bacterial genera or genus-like cluster of uncultured *Bacteria* (genus-level taxonomic path, SILVA phylogeny) in whale faecal samples. Depicted values represent relative abundances given in percent (%). Samples are sorted according to whale genera and species. Most abundant genera are given at the top. Mean and standard deviations (SD) as relative abundances for the two whale families. Each detected genus was assigned with a unique tax-ID (T-number). Heatmap colors indicate relative abundance. SIMPER analysis indicates the contribution of individual phyla on differences among the composition of the faecal microbiomes of the investigated baleen and toothed whales. T-tests indicate if differences in relative abundance of individual genera in baleen and toothed whales were significant (p<0.05; labelled in yellow).

**Supplementary Table S7** Alpha diversity estimates for individual whale feces microbiomes performed at the genus level. Analysis was conducted in PAST4 and based on the number of genera/genus-like cluster (genus-level taxonomic path) and the number of sequences per genus-level taxonomic path. T-tests indicate if differences in alpha diversity parameters were significant among baleen and toothed whales (p<0.05).

|  | Taxa_S | Chao-1 | Evenness_e^H/S | Dominance_D | Shannon_H | Simpson_1-D |
| --- | --- | --- | --- | --- | --- | --- |
| Bbo036 | 150 | 202.1 | 0.03065 | 0.4473 | 1.526 | 0.5527 |
| Bmu018 | 179 | 239.2 | 0.03815 | 0.3674 | 1.921 | 0.6326 |
| Bmu025 | 199 | 243.5 | 0.07842 | 0.1253 | 2.748 | 0.8747 |
| Bph044 | 184 | 235 | 0.04616 | 0.3233 | 2.139 | 0.6767 |
| Bph048 | 192 | 229.1 | 0.1208 | 0.08645 | 3.144 | 0.9135 |
| Bph049 | 183 | 201.2 | 0.06832 | 0.2061 | 2.526 | 0.7939 |
| Bph050 | 201 | 244.2 | 0.1092 | 0.09699 | 3.089 | 0.903 |
| Bph053 | 148 | 199.2 | 0.1506 | 0.08491 | 3.104 | 0.9151 |
| Bph055 | 178 | 231.7 | 0.03211 | 0.4328 | 1.743 | 0.5672 |
| Bph057 | 211 | 254.1 | 0.1354 | 0.0772 | 3.352 | 0.9228 |
| Bph058 | 205 | 252.8 | 0.1266 | 0.08331 | 3.256 | 0.9167 |
| Bph063 | 223 | 272 | 0.1199 | 0.07459 | 3.286 | 0.9254 |
| BphF003 | 182 | 197 | 0.07296 | 0.2213 | 2.586 | 0.7787 |
| Pma179 | 145 | 193.5 | 0.1349 | 0.0838 | 2.974 | 0.9162 |
| Pma185 | 211 | 258.3 | 0.1115 | 0.09007 | 3.158 | 0.9099 |
| Pma188 | 125 | 140 | 0.09282 | 0.2514 | 2.451 | 0.7486 |
| Pma190 | 186 | 230.4 | 0.1377 | 0.08157 | 3.243 | 0.9184 |
| t-test (baleen vs. toothed whales)  p-values | 0.188 | 0.200 | 0.033 | 0.111 | 0.123 | 0.111 |

**Supplementary Table S8** Relative abundance of bacterial genera/ genus-like cluster (genus-level taxonomic path, SILVA phylogeny) in whale faecal samples by the *Archaea* specific 16S rRNA gene amplicon sequencing approach. Depicted values represent relative abundances given in percent (%). Samples are sorted according to whale genera and species.

**Supplementary Figures**

**
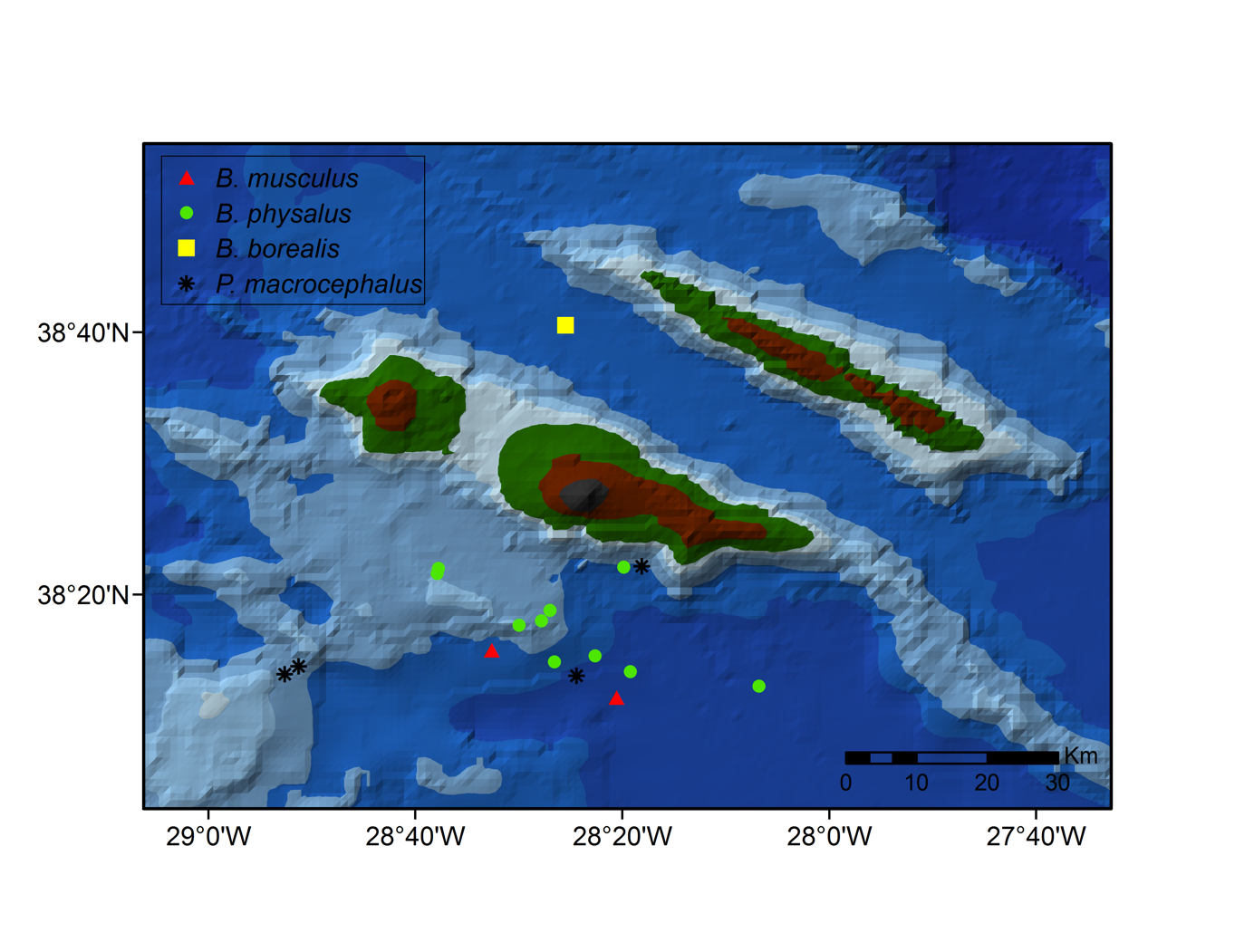
**

**Supplementary Figure S1** Overview of the whale faeces sampling points

**Supplementary Fig. S2** Quality control of DNA extracts obtained from ethanol stored fecal samples of whales. **a**, Ethidium bromide stained PCR products resolved at a 1.4% agarose gel after amplification from whale fecal DNA extracts with universal 16S rRNA gene targeting primers GC339F/907R. **b**, Bacterial community fingerprint patterns generated by DGGE analysis of from PCR amplified 16S rRNA gene fragments (a) representing the dominating bacterial communities in whale faecal samples. NTC: non template control; S: standards in a: 100 bp DNA ladder (Thermo Scientific, formerly Fermentas), b: DNA standard representing a mixture of PCR amplified 16S rRNA gene fragments of different bacterial type strains (according to Schellenberg et al*.* 2020).

**Supplementary Fig. S3** Statistical analysis of bacterial community fingerprint patterns of the abundant whale feces microbiota generated by PCR-DGGE analysis as depicted in Supplementary Fig. S2. **a**, UPGMA clustering of bacterial community fingerprint patterns based on a Pearson correlation based similarity matrix. Analysis was performed in GelCompar II [Applied Maths according to Schellenberg et al. (2020)]. **b**, NMDS plot based on the comparison of bacterial community fingerprint patterns by Bray Curtis distances. Analysis was performed in PAST version 3.11 using relative band surface pattern extracted from GelCompar II. Barleen and toothed whales are grouped respectively. One Way ANOSIM analysis (based on Bray Curtis distances; 9999 permutations N) confirmed significant differences among baleen and toothed whale community fingerprint patterns (Bonferroni corrected p-value = 0.0005).

**Supplementary Fig. S4** Rarefaction curves of bacterial 16S rRNA gene Illumina based amplicon data. Rarefaction curves based on the number of genera (genus-level taxonomic path) and number of 16S rRNA gene amplicon sequences per phylogenetic group. Analysis was performed in PAST4.
